# Colorectal Endoscopic Submucosal Dissection Using a Single Multifunctional Knife With a High‐Pressure Waterjet System

**DOI:** 10.1111/den.70164

**Published:** 2026-05-08

**Authors:** Koyo Kido, Yohei Ikenoyama, Hayato Nakagawa

**Affiliations:** ^1^ Department of Gastroenterology and Hepatology Mie University Hospital Mie Japan

## 1 Brief Explanation

**FIGURE 1:**
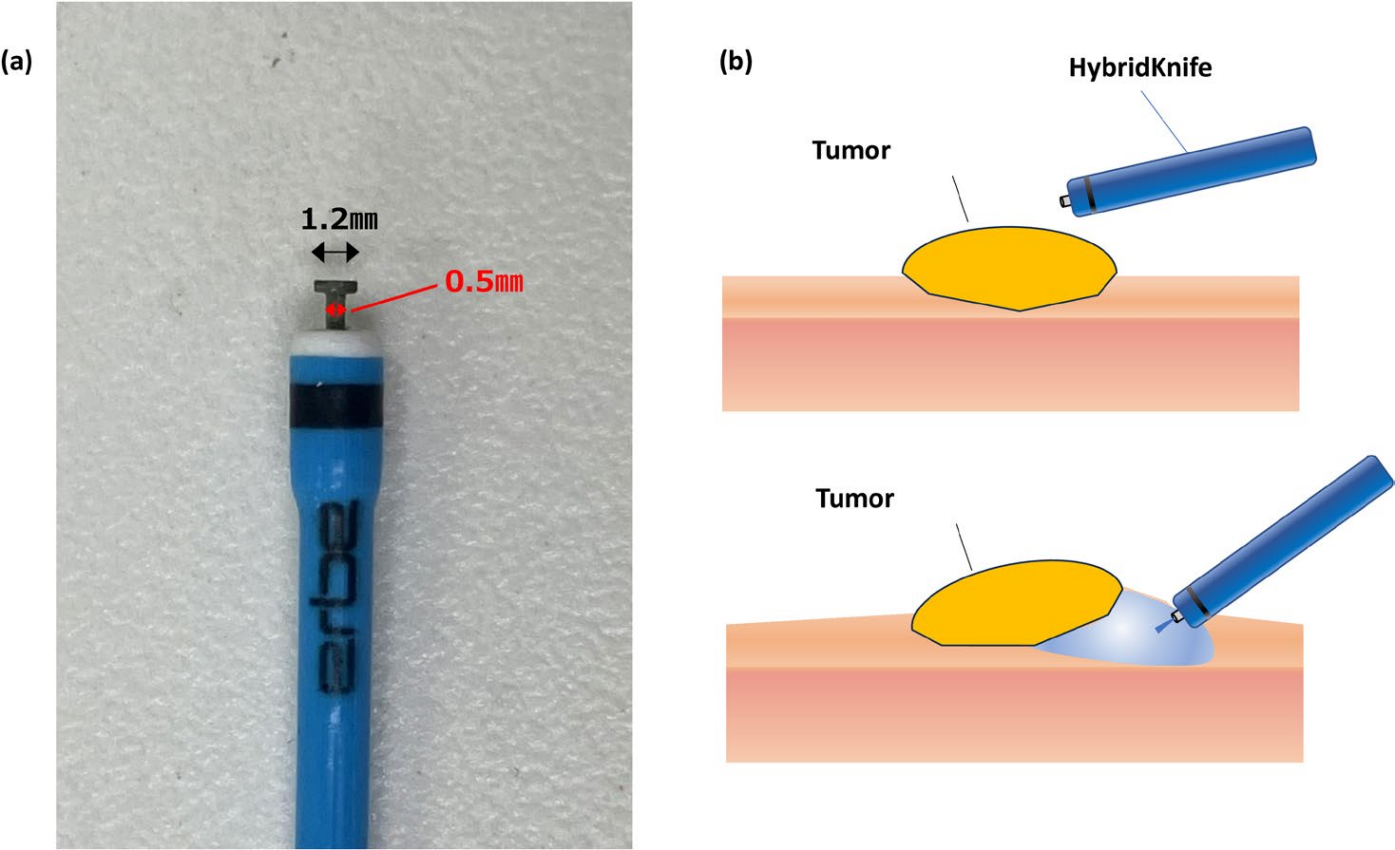
Schema of HybridKnife flex. (a) An enlarged image of the HybridKnife flex T‐type (Erbe Elektromedizin, Tübingen, Germany). High‐pressure injection can be performed directly from the knife tip. Compared with the conventional HybridKnife, the knife tip diameter of the HybridKnife flex has been reduced from 0.7 to 0.5 mm, enabling more delicate and precise manipulation. (b) The combination of the HybridKnife and the ERBEJET 2 high‐pressure waterjet system (Erbe Elektromedizin, Tübingen, Germany) enables sufficient submucosal injection without any need for a needle.

Endoscopic submucosal dissection (ESD) is a well‐established procedure. The Hybridknife (Erbe, Tübingen, Germany) enables submucosal injection, incision, and dissection using a single device [1]. Recently, a new‐generation Hybridknife flex has been designed with the expectation of improving injection and hemostatic performance based on preclinical evaluation [2, 3] (Figure 1). We report a case of colorectal ESD performed using the Hybridknife flex.

**FIGURE 2:**
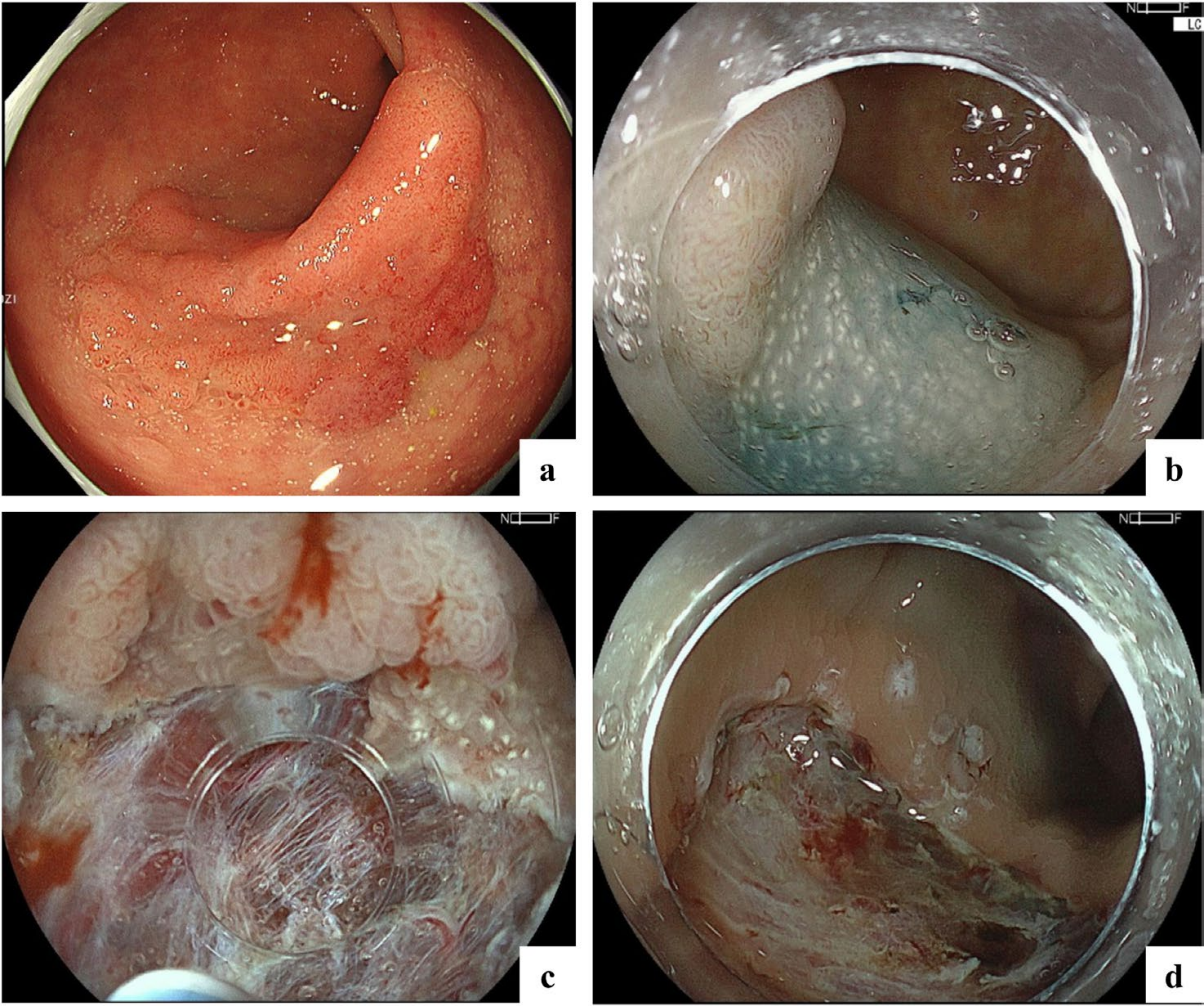
(a) A 40‐mm laterally spreading tumor of the granular homogeneous type (LST‐G, Homo type) was located on the rectum (Rs). (b) Sufficient submucosal injection can be achieved without incision. (c) Despite mild fibrosis, submucosal injection could be maintained throughout the dissection without changing devices. (d) All procedures were performed using a single device; en bloc resection was performed without any adverse events.

A woman in her 60s presented with a 40‐mm laterally spreading granular‐type tumor in the rectosigmoid colon (Figure 2a). ESD was performed using the Hybridknife flex T‐type with a 1.5‐mm tip (Erbe, Tübingen, Germany), combined with the needleless high‐pressure waterjet system (ERBEJET 2, Effect;40). An endoscope (EG‐760Z; Fujifilm, Tokyo, Japan) with a transparent distal hood was used with an EP‐8000 (Fujifilm, Tokyo, Japan) as the light source. The electrosurgical settings were EndoCut U mode (Effect 1, Duration 4, and Interval 2) and precise SECT mode (Effect 3.5) using a VIO 3 electrosurgical generator (Erbe). A high‐pressure submucosal injection of saline containing indigo carmine was administered through the device tip to provide sufficient submucosal elevation (Figure 2b), without prior needle injection. Despite slight fibrosis, the submucosal layer could be clearly visualized (Figure 2c). All procedures were performed using a single device; en bloc resection was performed without any adverse events (Figure 2d). Pathology revealed an intramucosal, well‐to‐moderately differentiated adenocarcinoma, with negative margins and no lymphovascular invasion (Video 1).

**VIDEO 1:** Colorectal endoscopic submucosal dissection using the HybridKnife® flex. Video content can be viewed at https://onlinelibrary.wiley.com/doi/10.1111/den.70164.

The Hybridknife flex is a tip‐type water‐jet knife that allows direct injection from the knife tip and favorable injection suggested in an experimental study [4]. This case showed that this device provided submucosal elevation without prior needle injection, and no injection‐related bleeding was observed. These advantages may eliminate the need for an injection needle, potentially shortening procedure time and reducing device exchanges.

## Funding

The authors have nothing to report.

## Disclosure

The authors have nothing to report.

## Conflicts of Interest

The authors declare no conflicts of interest.

## Data Availability

The data that support the findings of this study are available on request from the corresponding author. The data are not publicly available due to privacy or ethical restrictions.
